# Leptin receptor is expressed by tissue mast cells

**DOI:** 10.1007/s12026-018-9029-0

**Published:** 2018-09-29

**Authors:** Paulina Żelechowska, Magdalena Wiktorska, Sylwia Różalska, Olga Stasikowska-Kanicka, Małgorzata Wągrowska-Danilewicz, Justyna Agier, Ewa Brzezińska-Błaszczyk

**Affiliations:** 1 Department of Experimental Immunology, Medical University of Lodz, Lodz, Poland; 2 Department of Molecular Cell Mechanisms, Medical University of Lodz, Lodz, Poland; 3 Department of Industrial Microbiology and Biotechnology, University of Lodz, Lodz, Poland; 4 Department of Nephropathology, Medical University of Lodz, Lodz, Poland

**Keywords:** Mast cells, Adipocytokines, Leptin, Leptin receptor, Ob-R

## Abstract

Leptin, the adipose tissue-derived product of the obese (*ob*) gene, is known to function as the hormone of energy expenditure. It has also been established that leptin regulates immune and inflammatory processes. All leptin-induced biological activities depend on binding to the membrane-spanning leptin receptor (Ob-R), belonging to the class I cytokine receptor family. The available data relating to the Ob-R on mature mast cells (MCs), and consequently leptin significance in the modulation of MC activity within the tissue, are limited. Immunohistochemistry was used to establish Ob-R expression by MCs in the mesenteric adipose tissue. Flow cytometry and confocal microscopy were used to evaluate both constitutive and leptin-induced expression of Ob-R on freshly isolated peritoneal MCs. MCs in the mesenteric adipose tissue and native peritoneal MCs express Ob-R constitutively. Additionally, leptin influences its receptor expression on these cells. Leptin at lower concentrations caused Ob-R expression increase both at the cell surface and in the cell interior. MC stimulation with higher concentrations of leptin results in a decline of Ob-R from the cell surface and significant enhancement of this receptor not only in the nuclear region but also in the endoplasmic reticulum. In conclusion, one can be assumed that leptin regulates MC activity within tissues. These findings might provide an additional link among the leptin, innate immune function, and inflammatory processes and diseases.

## Introduction

Among the adipocytokines, leptin, a 16-kDa non-glycosylated polypeptide product of the obese (*ob*) gene, has received particular attention. Even though this adipocytokine is secreted predominantly by adipocytes, it is also synthesized by placenta trophoblast, bone cells, chondrocytes, skeletal muscle cells, gastric chief cells, and various immune cells, such as T cells, basophils, and mast cells (MCs) [2, 10, 14, 29]. Leptin is primarily recognized for its role as a hypothalamic modulator of food intake, body weight, and energy expenditure [21]. However, some subsequent studies identified leptin as a versatile molecule exerting diverse effects on angiogenesis, hemopoiesis, reproduction, and neuroendocrine system. More importantly, it has been established that leptin regulates immune and inflammatory processes [24, 25]. Specifically, leptin promotes the expression of endothelial cell adhesion molecules and has the potency to recruit immune cells including monocytes/macrophages, neutrophils, eosinophils, basophils, endothelial, and dendritic cells. In monocytes and macrophages, leptin upregulates phagocytic activity and the release of oxygen radicals [8]. Apart from the above described, leptin stimulates immune cells such as monocytes/macrophages, eosinophils, basophils, NK, and dendritic cells to the pro-inflammatory mediator production [12].

All leptin-induced biological activities depend on binding to the membrane-spanning leptin receptor (Ob-R), belonging to the class I cytokine receptor family. This adipocytokine binds to Ob-R and initiates downstream signaling through the phosphorylation of the tyrosine kinase Janus kinase 2 (JAK2) and the transcription factor signal transducer and activator of transcription (STAT). The Ob-R expression is not limited to the hypothalamic neurons, astrocytes, or microglia cells [15, 17, 30]. It is also found in peripheral tissues and different cell populations of the immune system, i.e., B and T cells, NK cells, macrophages/monocytes, neutrophils, basophils, eosinophils, epithelial, and dendritic cells [12].

MCs are long-lived tissue resident cells, which are distributed throughout all organs and connective tissue types. It should be mentioned that these cells are also present in the adipose tissue, next to adipocytes. The cytoplasm of MCs contains 50–200 large granules that store preformed granule-associated mediators (i.e., histamine, chymase, tryptase, metalloproteinases, and proteoglycans). Moreover, MCs are capable of producing a wide array of newly generated lipid mediators, such as leukotrienes (LTs), prostaglandins (PGs), thromboxanes (TXs), and platelet-activating factor (PAF), as well as de novo synthesized cytokines and chemokines [20]. Therefore, apart from being markedly involved in allergic reactions, MCs are critical players for the maintenance of body homeostasis by acting on wound healing, angiogenesis, and vascular permeability. Undoubtedly, MCs are implicated in a variety of pathological conditions and strongly influence both innate and acquired immune responses. These cells are known to be enrolled in host defense against pathogens and play an essential role in both acute and chronic inflammation as well [11, 22]. As MCs may modulate the course of inflammation in different ways, they play a significant role in inflammatory processes and diseases. They are involved, for example, in the “low-grade inflammation” within adipose tissue, a state accompanying obesity [31], as well as in neuroinflammation [6] and fibromyalgia [18].

Since MCs are strongly involved in the course of immunological and inflammatory processes, and leptin affects both of them, it seems to be of great importance to understand the impact of leptin on MC biology. Therefore, we decided to examine the Ob-R expression by mature tissue MCs. The available data concerning the Ob-R presence in these cells, and consequently, leptin significance in the modulation of MC activity within the tissue are limited. Up to date, the Ob-R has been identified in murine bone marrow-derived MCs (BMMCs) [32] and in MCs in human skin, respiratory, gastrointestinal, and urogenital tracts [29]. Immunohistochemistry was used to establish Ob-R expression by MCs in the mesenteric adipose tissue. By flow cytometry and confocal microscopy, we conducted analyses of Ob-R expression on matured in vivo rat MCs isolated from the peritoneal cavity, that is, connective tissue-type MCs. We also scheduled to establish whether leptin by itself affects the expression of its receptor in mature MCs.

## Materials and methods

### Materials

SuperFrost Plus slides were purchased from Gerhard Menzel GmbH (Braunschweig, Germany). Target retrieval solution (TRS), Tris-buffered saline (TBS), EnVision+System-HRP, and 3,3′-diaminobenzidine were obtained from Dako Corp. (Carpinteria, USA). Dulbecco’s modified eagle medium (DMEM) was purchased from Biowest (Kansas City, USA). Hank’s balanced salt solution (HBSS), sodium bicarbonate, fetal calf serum (FCS), gentamicin, and glutamine were obtained from GIBCO (Gaithersburg, USA). Hydrogen peroxide solution, NaCl, DPX mountant for histology, Percoll®, hematoxylin, toluidine blue, trypan blue, and phosphate-buffered saline (PBS) were purchased from Sigma-Aldrich (St. Louis, USA). Recombinant rat leptin and normal rabbit IgG antibodies were obtained from R&D Systems (Minneapolis, USA). BD CellFIX™ was obtained from BD Biosciences (Benelux, Belgium). Rabbit polyclonal IgG antibodies against Ob-R were purchased from Thermo Fischer Scientific (Waltham, USA). Alexa Fluor® 488-conjugated goat anti-rabbit polyclonal antibodies were obtained from Jackson ImmunoResearch Laboratories, Inc. (West Grove, USA).

### Animals

The study was performed on female albino Wistar rats weighing ~ 250 g, aged 3 to 4 months. The animals were obtained from the animal quarters of the Faculty of Biology and Environmental Protection of the University of Lodz. Standard storage conditions were provided: the animals were kept in metal cages, five rats in each, at room temperature. They were maintained under artificial lighting for a 12-h light cycle. The animals were fed with Murigran granulated fodder for rodents and water ad libitum. Isoflurane-induced anesthesia was carried out before animal decapitation. All efforts were made to minimize animal suffering. Protocols were approved by the Local Ethics Committee for Experiments on Animals in Lodz (the approval number 55/ŁB42/2016).

### Immunohistochemistry and toluidine blue staining

Sections of mesenteric adipose tissue were collected from the rats. Staining procedures were performed on the two serial paraffin-embedded tissue sections of the mesenteric adipose tissue. First, paraffin-embedded tissue section was mounted onto SuperFrost slides, deparaffinized with xylene, then treated in a microwave oven in a TRS (pH 6.0) for 30 min (2 × 5 min at 360 W, 2 × 5 min at 180 W, and 2 × 5 min at 90 W) and transferred to distilled water. Endogenous peroxidase activity was blocked by 0.3% hydrogen peroxide in distilled water for 30 min. Then section was rinsed with TBS and incubated for all night in the humidified atmosphere with polyclonal rabbit anti-Ob-R antibody (dilution, 1:400). Afterward, EnVision+System-HRP prepared according to the instructions of the manufacturer was used. Visualization was performed by incubating the sections in a solution of 3,3′-diaminobenzidine. After washing, the section was counter-stained with hematoxylin and coverslipped. The negative control was carried out by incubation in the absence of the primary antibody and always yielded negative results.

Second, paraffin-embedded tissue section was mounted onto slides, deparaffinized with xylene, then incubated for 3 min with toluidine blue solution (1 g toluidine blue dissolved in 70% ethanol) in 10% NaCl solution, washed three times in distilled water, dehydrated, and mounted with DPX mountant for histology. In the tissue sections of mesenteric adipose tissue, the number MCs stained with toluidine blue and immunopositive cells was on the similar level.

### Isolation of peritoneal MCs

Peritoneal cell suspensions were obtained from the peritoneal cavities by lavage with 50 ml of 1% HBSS supplemented with 0.015% sodium bicarbonate. After the abdominal massage, the cell suspension was removed from the peritoneal cavity, centrifuged (1200 rpm, 5 min, 20 °C), and washed twice in complete (c)DMEM containing DMEM supplemented with 10% FCS, 2 mM glutamine, and 10 μg/ml gentamicin. To obtain purified MCs, the cell suspensions were resuspended in 72.5% isotonic Percoll® and centrifuged (1500 rpm, 15 min, 20 °C). The upper cell layer was removed, and pelleted MCs were washed twice in cDMEM by centrifugation (1200 rpm, 5 min, 20 °C). After washing, MCs were counted and resuspended in an appropriate volume of cDMEM, to obtain an MC concentration of 1.5 × 10^6^ cells/ml. MCs were prepared with purity > 98%, as determined by metachromatic staining with toluidine blue. The viability of MCs was over 98%, as estimated by trypan blue exclusion method.

### Cell preparation for flow cytometric and confocal microscopy analysis

Constitutive and leptin-induced Ob-R expression was determined using flow cytometry and confocal microscopy. Constitutive expression of Ob-R was assessed in freshly isolated native MCs (NS, non-stimulated cells). Induced receptor expression was estimated in MCs incubated with leptin, at the final concentrations of 0.1, 1, 10, or 100 ng/ml, for 1 h at 37 °C in a humidified atmosphere with 5% CO_2_. Following this, the MCs were fixed with CellFIX solution for 15 min at 4 °C and washed twice with 1× PBS. To determine the intracellular localization of Ob-R, the MCs were permeabilized with 0.1% saponin for 30 min at room temperature. Next, MCs were resuspended in 1× PBS and stained for 1 h with rabbit polyclonal anti-Ob-R antibodies (dilution, 1:100). For control, MCs were stained with rabbit IgG isotype control with irrelevant specificity. Primary antibody was not added to the sample to certify non-specific binding of the secondary antibody. Cells were then washed with 1× PBS and incubated with Alexa Fluor® 488-conjugated secondary antibodies (dilution, 1:100) in 1× PBS for 1 h in the dark. Following this, the cells were washed twice and finally resuspended in 1× PBS before receptor assessment. After each period of incubation, MC viability was examined using the trypan blue exclusion test.

### Flow cytometry

Ten thousand events in each sample were analyzed using a FACSCalibur flow cytometer with CellQuest software (BD Biosciences). Leptin-dependent MC Ob-R expression was presented as a percentage of Ob-R MFI (mean fluorescence intensity) measured in native MCs (referred to as 100%).

### Confocal microscopy

The samples were mounted on microscope slides, and images were captured using an LSM 510 meta confocal laser scanning microscope (Zeiss, Oberkochen, Germany) combined with an Axi1overt 200 M (Zeiss) inverted microscope equipped with a Plan-Neofluar objective (40×/0.6). All settings were held constant throughout the experiments except for gain factor, which was adjusted for each receptor. The fluorescence was recorded using the argon laser (488 nm) and a BP filter set (505 nm). The same laser line was used for Nomarski DIC. All signals obtained from confocal microscopy were validated with profile view image analysis and the diagrams presenting intensity values placed beside each microphotograph. The mean fluorescence intensity (expressed in arbitrary units AU) was calculated for each of the samples. The calculations were performed for at least 40 different points randomly selected in compartments with receptor expression.

### Statistical analysis

The statistical analysis of the experimental data was performed using Statistica 13 software (Statsoft Inc., USA). Data are presented as mean ± SD. Normality of distribution was tested with the Shapiro-Wilk test. All comparisons between groups were carried out by using Student’s *t* test for small groups. Differences were considered significant at *P* < 0.05 and are labeled with an asterisk (*) on each graph.

## Results

### Histochemical imaging of MCs in mesenteric adipose tissue

**Fig. 1:**
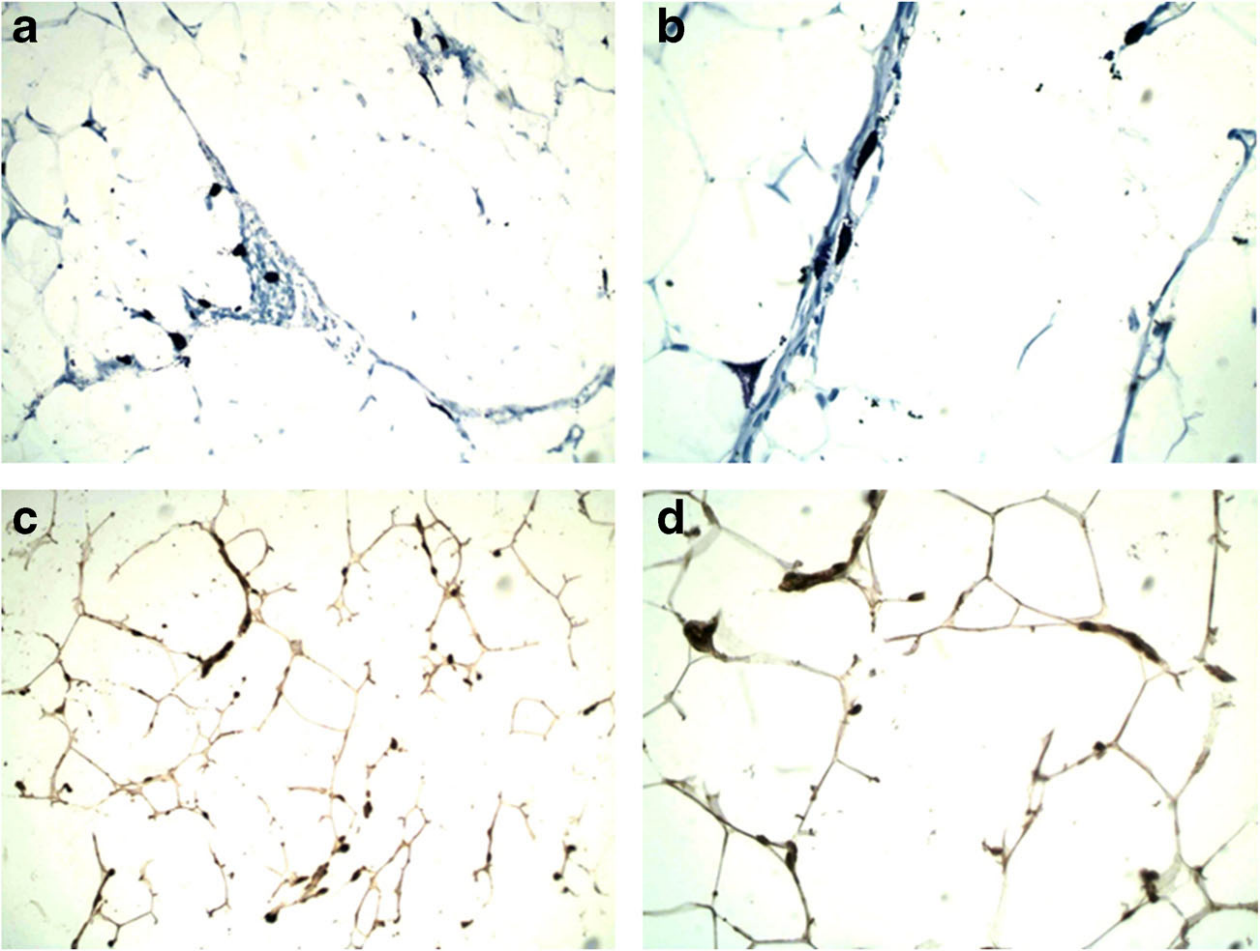
MCs in the mesenteric adipose tissue. Metachromatic toluidine blue staining; magnifications × 200 (**a**) and × 400 (**b**). Ob-R immunopositive MCs; magnifications × 200 (**c**) and × 400 (**d**)

Toluidine blue staining revealed that MCs were scattered throughout the mesenteric adipose tissue. These cells were relatively large and pleomorphic and their shape varied from round or oval to extraordinarily elongated or star-shaped forms. Oval to roundish nuclei were visible in cells if not covered by granules. Some of MCs were degranulated (Fig. 1a, b). Immunohistochemistry was used to evaluate the Ob-R expression by MCs within this tissue. A subset of cells immunopositive for Ob-R was localized similarly to cells stained with toluidine blue. Moreover, the morphologic picture of these cells resembled MCs stained with toluidine blue. The pattern of immunopositivity for Ob-R in MCs was cytoplasmic (Fig. 1c, d).

### Constitutive expression of Ob-R by native MCs

**Fig. 2:**
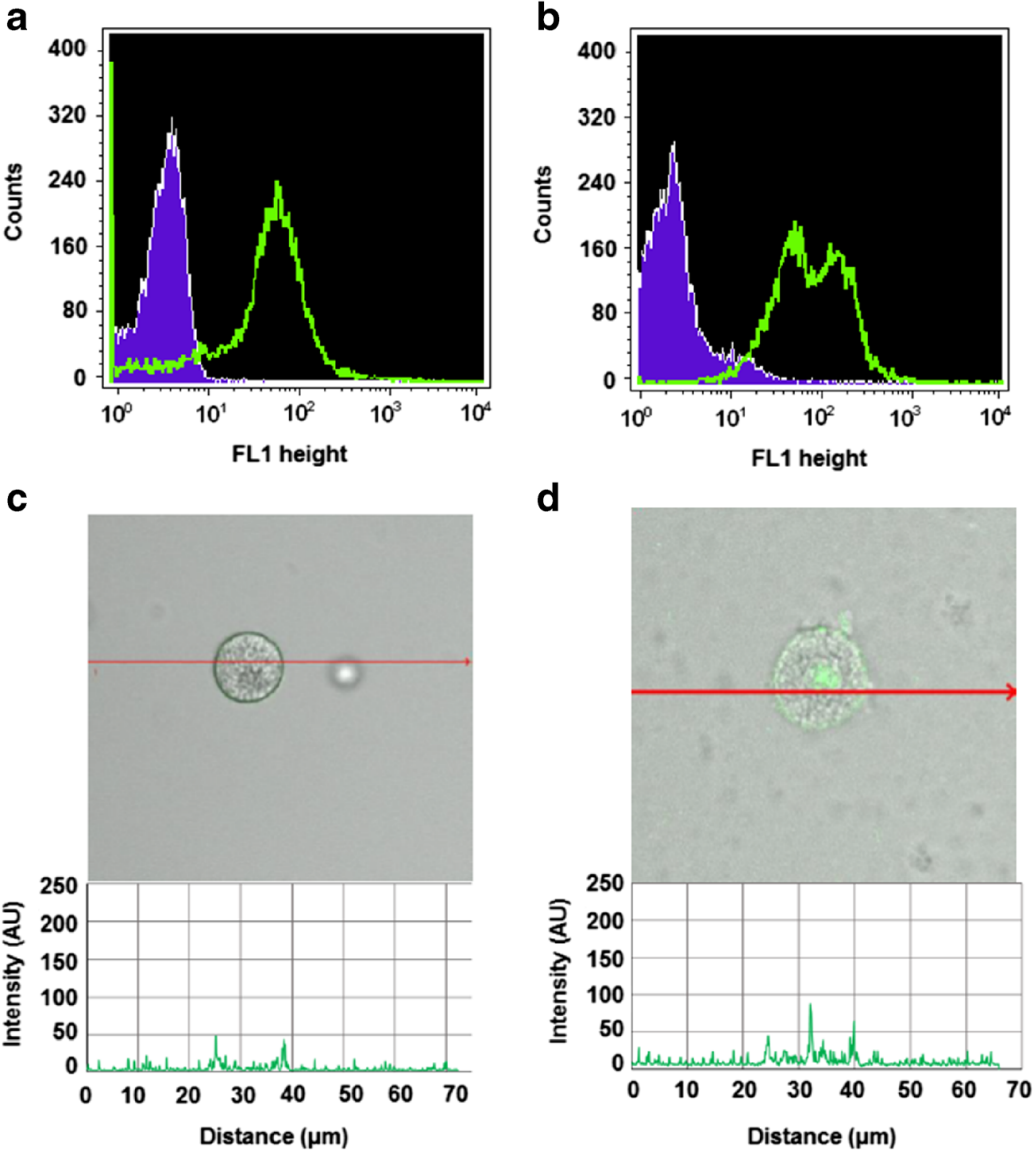
Constitutive expression of Ob-R in mature rat peritoneal MCs analyzed by flow cytometry (**a**, **b**) (shaded tracings, isotype control; open tracings, Ob-R expression) and by confocal microscopy (**c**, **d**). Fluorescence intensity diagrams below each microphotograph showing the distribution of fluorescence in cells were mounted. Surface Ob-R expression (a, c) and intracellular Ob-R expression (b, d). The results shown are representative of three independent experiments performed in duplicate. The signal was visualized with green Alexa 488

Flow cytometry technique was used to determine the constitutive expression of Ob-R by freshly isolated native tissue rat MCs. Staining of non-permeabilized (Fig. 2a) and permeabilized (Fig. 2b) MCs showed that Ob-R protein was located both at the cell surface and intracellularly. To assess the cellular distribution of Ob-R, confocal microscopy was used. The fluorescence intensity diagram beside microphotograph shows signals from the cell surface of native non-permeabilized MCs (Fig. 2c). A confocal microscopy image analysis also confirmed the intracellular presence of Ob-R in native permeabilized MCs. The signals were associated mainly with the nuclear region, as seen in the fluorescence intensity diagram (Fig. 2d). Isotype controls and controls for non-specific binding of the secondary antibody confirmed the specificity of antibodies (data not shown).

### Effect of leptin on surface Ob-R expression in MCs

**Fig. 3:**
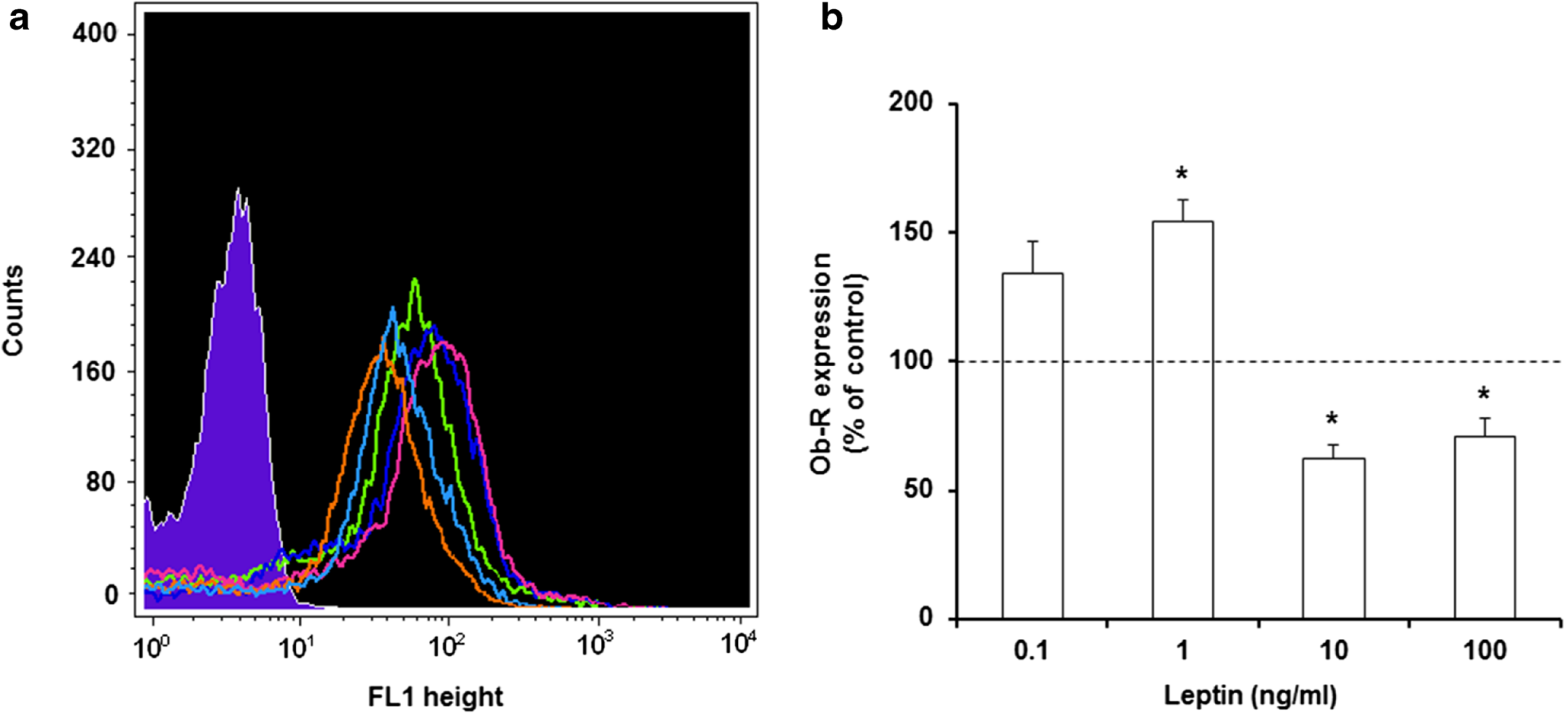
Flow cytometric analysis of surface Ob-R expression in MCs stimulated by leptin. Representative flow cytometry histogram showing surface Ob-R expression (**a**): shaded tracing, isotype control; open tracings, Ob-R expression in unstimulated cells (green), and in cells stimulated with leptin at concentrations of 0.1 ng/ml (dark blue), 1 ng/ml (red), 10 ng/ml (orange), and 100 ng/ml (light blue). Expression levels of surface Ob-R in unstimulated and leptin-stimulated MCs (**b**). Constitutive Ob-R expression served as a control and was referred to as 100%. The results are presented as a percentage of constitutive Ob-R expression and are the means of fluorescent intensity ± SD of three independent experiments performed in duplicate. The signal was visualized with green Alexa 488. *P < 0.05

The impact of leptin stimulation on surface Ob-R expression was evaluated by flow cytometry in MCs exposed to this adipocytokine at concentrations of 0.1, 1, 10, or 100 ng/ml for 1 h (Fig. 3). After MC stimulation with 0.1 ng/ml of leptin, irrelevant increase in cell surface Ob-R expression level compared with the control expression in unstimulated MCs was observed. The baseline expression level of surface Ob-R was significantly (*P* < 0.05) upregulated following incubation with leptin at a concentration of 1 ng/ml reaching 153.0 ± 8.5% of control Ob-R expression in native MCs. Stimulation with leptin used at a concentration of 10 ng/ml resulted in a statistically significant (*P* < 0.05) decrease of Ob-R surface expression level-up to 62.1 ± 5.6% of control. Similarly, MC activation with 100 ng/ml of leptin caused considerable down-regulation of Ob-R expression (up to 70.5 ± 7.4% of control).

**Fig. 4:**
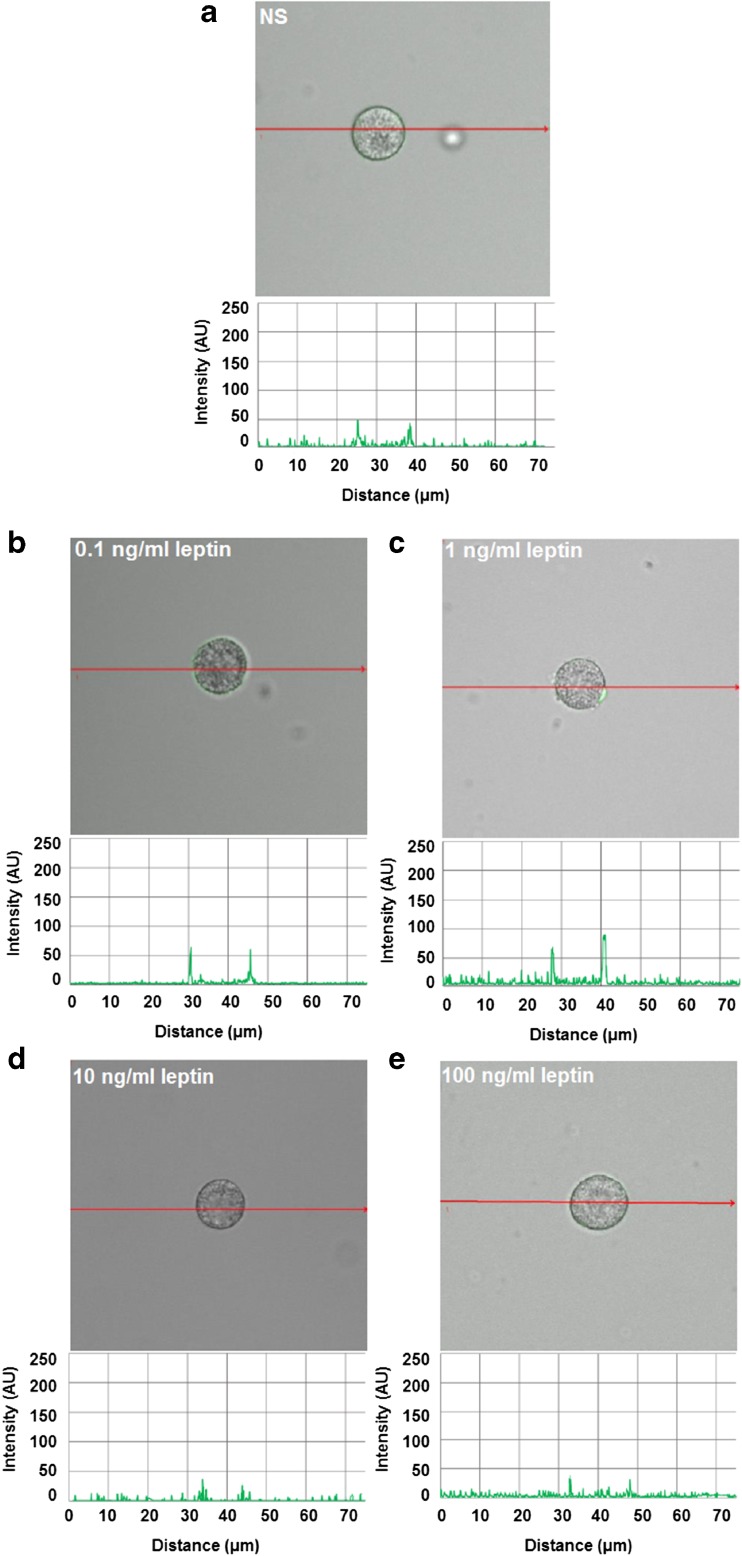
Confocal microscopy images of surface Ob-R expression in MCs stimulated by leptin. Cells were incubated with medium alone (unstimulated cells; NS) (**a**), leptin at concentrations of 0.1 (**b**), 1 (**c**), 10 (**d**), or 100 ng/ml (**e**). Single confocal sections (midsection of cells) showing the presence of Ob-R and fluorescence intensity diagrams beside microphotographs showing the distribution of fluorescence in cells. Data shown are from one representative experiment out of three performed in duplicate. The signal was visualized with green Alexa Fluor 488

These findings are in good agreement with the confocal microscopy analysis (Fig. 4). The fluorescence intensity diagram beside microphotograph showed that when MCs were stimulated with 0.1 ng/ml of leptin, cell surface signals were insensibly increased as compared with native cells (31.2 ± 19.3 AU vs. 20.2 ± 8.0 AU) (Fig. 4a, b). After MC stimulation with 1 ng/ml of leptin, image analysis revealed that intensity of cell surface fluorescence was substantially higher (60.0 ± 24.0 AU) in comparison to unstimulated cells (Fig. 4c). In turn, confocal and fluorescence intensity images showed that MC treatment with higher leptin concentrations, i.e., 10 or 100 ng/ml induced a decline of Ob-R expression from the cell surface of MCs as compared to unstimulated cells (Fig. 4d, e).

### Effect of leptin on intracellular Ob-R expression in MCs

**Fig. 5:**
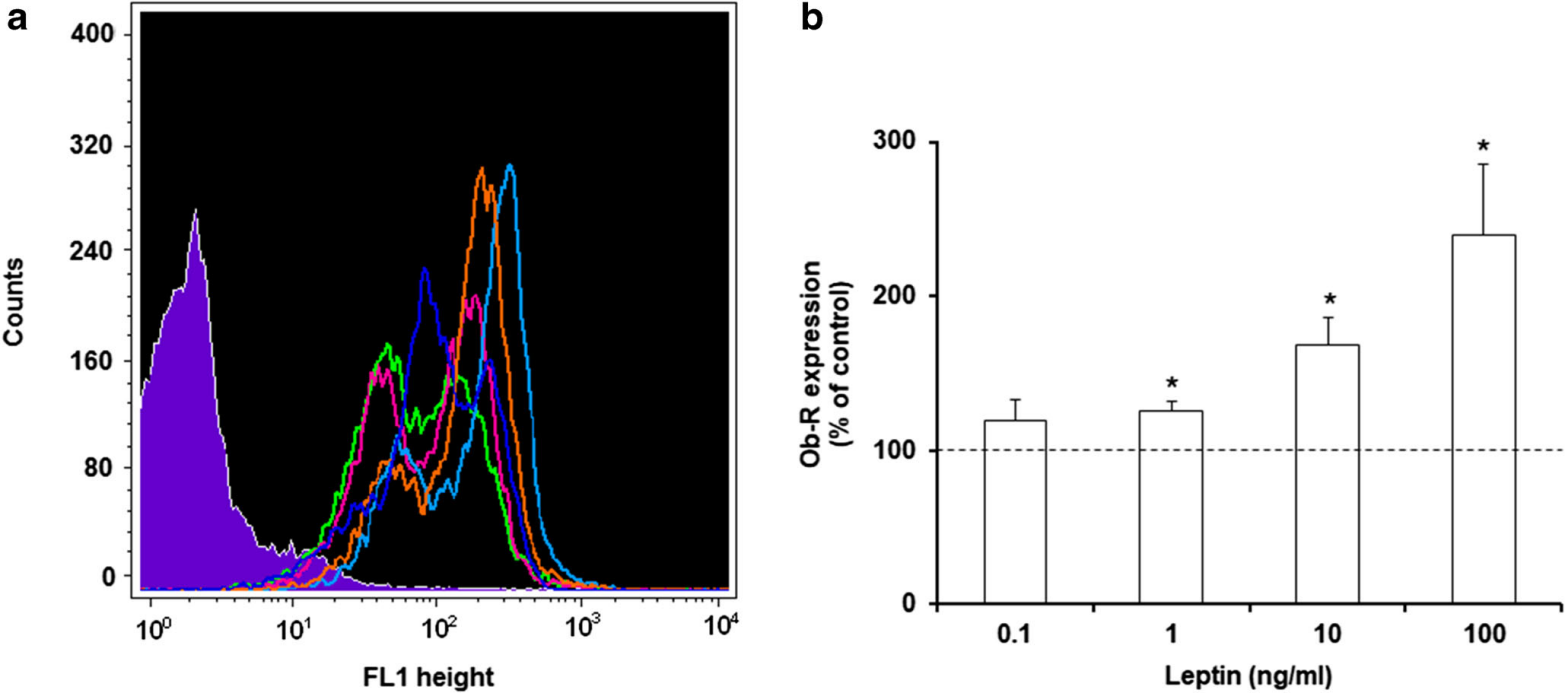
Flow cytometric analysis of intracellular Ob-R expression in permeabilized MCs stimulated by leptin. Representative flow cytometry histogram showing intracellular Ob-R expression (**a**): shaded tracing, isotype control; open tracings, Ob-R expression in unstimulated cells (green), and in cells stimulated with leptin at concentrations of 0.1 ng/ml (dark blue), 1 ng/ml (red), 10 ng/ml (orange), and 100 ng/ml (light blue). Expression levels of intracellular Ob-R in unstimulated and leptin-stimulated MCs (**b**). Constitutive Ob-R expression served as a control and was referred to as 100%. The results are presented as a percentage of constitutive Ob-R expression. Results are the mean of fluorescent intensity ± SD of three independent experiments performed in duplicate. The signal was visualized with green Alexa Fluor 488. *P < 0.05

The intracellular expression of Ob-R by permeabilized MCs is shown in Fig. 5. After MC stimulation with 0.1 ng/ml of leptin, we observed no statistically significant increase in intracellular Ob-R expression level. The baseline level of Ob-R expression was significantly up-regulated (*P* < 0.05) following 1 h incubation with leptin at concentrations of 1, 10 or 100 ng/ml, reaching 125.3 ± 6.5%, 167.8 ± 18.2%, and 239.0 ± 46.7% of control Ob-R expression in native MCs, respectively.

**Fig. 6:**
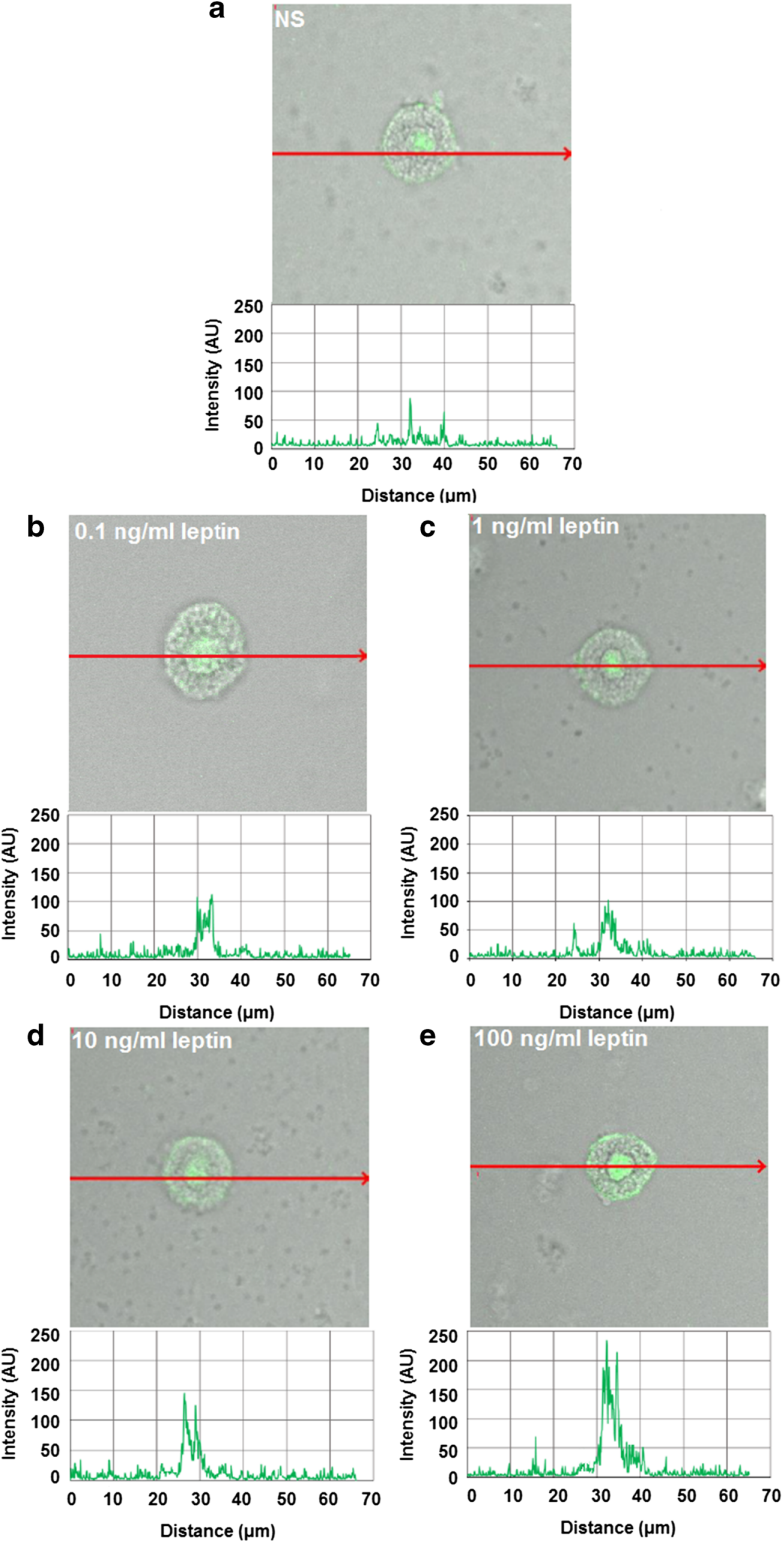
Confocal microscopy images of intracellular Ob-R expression in MCs stimulated by leptin. Cells were incubated with medium alone (unstimulated cells; NS) (**a**), leptin at concentrations of 0.1 (**b**), 1 (**c**), 10 (**d**), or 100 ng/ml (**e**). Single confocal sections (midsection of cells) showing the presence of Ob-R and fluorescence intensity diagrams beside microphotographs showing the distribution of fluorescence in cells. Data shown are from one representative experiment out of three performed in duplicate. The signal was visualized with green Alexa Fluor 488

Single confocal sections, supplemented with fluorescence intensity diagrams, showed that unstimulated permeabilized cells express Ob-R mainly in the nuclear region (Fig. 6a). MC stimulation with 0.1 ng/ml resulted in a slight increase of signals in nuclear region, but cell surface signals were comparable to signals obtained for unstimulated MCs which was documented by fluorescence intensity diagram beside microphotograph (Fig. 6b). After MC treatment with 1 ng/ml of leptin, significant receptor pool was associated with the nucleus, and slightly increase in fluorescence signals bound up with the cell surface was observed (Fig. 6c). MC stimulation with 10 and 100 ng/ml of leptin resulted in a decline of Ob-R from the cell surface (in comparison to the unstimulated cells) and significant enhancement of this receptor not only in the nuclear region but also endoplasmic reticulum as seen in intensity diagrams beside each microphotograph (Fig. 6d–e). Image analysis demonstrated that the baseline level of intracellular Ob-R expression in unstimulated cells (57.4 ± 27.2 AU) was upregulated upon incubation with leptin at a concentrations of 10 ng/ml (113.0 ± 14.6 AU) and 100 ng/ml (159.2 ± 34.6 AU).

## Discussion

There is only scarce information about the expression of Ob-R by MCs. Zhou et al. [32] noticed Ob-R expression in immature BMMCs. Taildeman et al. [29] documented the presence of this receptor in MCs in human tissue samples obtained from the skin, respiratory, gastrointestinal, and urogenital tracts. Immunofluorescence staining revealed predominant cytoplasmic and granular localization of Ob-R in human MCs; however, this leptin receptor was also expressed in the cytoplasmic rim lining the cell surface membrane. No nuclear localization of Ob-R in MCs was noticed. In the present study, we evaluated the expression of Ob-R in fully matured tissue rat MCs isolated from the peritoneal cavity. By the use of immunohistochemistry, flow cytometry technique, and confocal microscopy, we clearly documented that rat tissue MCs constitutively express Ob-R. Moreover, confocal microscopy analysis revealed that in native resting MCs, Ob-R is located not only on the cell surface but also within nuclear inclusions. Previously, Buyse et al. [5] documented that neurons express Ob-R protein located in the cytoplasmic compartments. The enterocytes show expression of Ob-R in the cytoplasm and basolateral membrane, as stated by immunohistochemistry technique [1]. Monocytes, neutrophils, eosinophils, and epithelial cells express Ob-R merely on the cell surface [4, 7, 9, 27].

Although different cell populations express Ob-R, data relating to the regulation of its expression are scarce. It was documented that interferon (IFN)-γ and interleukin (IL)-1β, but not tumor necrosis factor (TNF), upregulate Ob-R expression by epithelial cells [27], IL-33, but not IL-3, enhances expression of this receptor in basophils [28], and TNF increases Ob-R expression in different cell lines [13]. It is worth pointing out that all mentioned above cytokines strongly promote inflammatory processes. From a physiological point of view, however, it is very important whether Ob-R expression can be regulated by its ligand, namely leptin. It was shown that treatment of microglia cells with 1 μM of leptin resulted in Ob-R mRNA expression increase [30]. Suzukawa et al. [27] stated that in epithelial cells, Ob-R protein expression level is downregulated by 10 μM of leptin. It was also documented that stimulation of immature and mature dendritic cells with leptin at a concentration of 10 nM resulted in Ob-R mRNA and protein expression upregulation [19]. In this study, we evaluated, for the first time, the effect of leptin on its receptor expression in MCs. Confocal imaging indicated that lower concentrations of this adipocytokine caused Ob-R expression increase both at the cell surface and cell interior. Therefore, we hypothesized that increased expression of receptor protein might be the result of the synthesis of new molecules from the pool of mRNA present in the cells. MC treatment with higher leptin concentrations enhanced intracellular and decreased surface Ob-R expression causing a reduction in the number of Ob-R on the surface. It may, therefore, be suggested that this is a result of surface Ob-R internalization and desensitization of MCs to subsequent leptin stimulation. Furthermore, leptin in higher concentration may inhibit the ubiquitination and protein degradation process which proves the accumulation of the receptor inside the cells. Whatever it may be, the observation that higher leptin concentrations cause a decrease in cell membrane Ob-R expression can suggest a physiological feedback mechanism that protects against chronic and excessive receptor stimulation.

Up to date, the physiological levels of leptin in humans have not yet been precisely determined. However, previous studies have reported that blood plasma concentrations of leptin are 11.6 ng/ml in healthy individuals and 34.7 ng/ml in patients with obesity [26]. Bozan and Dogruel [3] noticed serum levels of leptin to be 7.82 ng/ml in non-obese and 72.1 ng/ml in obese children. Therefore, we may assume that leptin concentrations used in our study reflect their concentrations present in pathophysiological conditions.

In conclusion, MCs play a role in maintaining homeostasis, immunological reactions, host defense, and various pathophysiological processes. MCs also play a crucial role in host defense as they express pattern recognition receptors, for example, Toll-like receptors (TLRs), and they release pro-inflammatory and antimicrobial mediators [16]. Without a doubt, these cells are strongly involved in acute and chronic inflammatory reactions as well. It is also important to note that MCs occur in great number within adipose tissue, adjacent to leptin-produced adipocytes, and take part in low-grade inflammation development within adipose tissue in obese individuals [31]. Also, MCs strongly contribute to other inflammatory states like neuroinflammation [6] and fibromyalgia [18]. As MCs express Ob-R, and their expression is modulated by leptin, it is obvious that this adipokine, generally accepted as a pro-inflammatory mediator [23], could influence MC activity, especially in the course of inflammation. Moreover, MCs contain leptin within cytoplasmic granules [29] and therefore one can assumed that this adipokine could act both as a paracrine and autocrine regulator of MC activity. It should be also stressed that leptin by its own receptor influences the activity of many cells and therefore it modulates the course of different processes. Hence, the use of leptin or Ob-R antagonists could be a possible way to regulate various mechanisms, including those involving MC contribution. However, further studies are needed to clarify the influence of leptin on MC biology, and such experiments are now in progress in our laboratory.
